# Arbuscular mycorrhizal interactions of mycoheterotrophic *Thismia* are more specialized than in autotrophic plants

**DOI:** 10.1111/nph.14249

**Published:** 2016-10-14

**Authors:** Sofia I. F. Gomes, Jesús Aguirre‐Gutiérrez, Martin I. Bidartondo, Vincent S. F. T. Merckx

**Affiliations:** ^1^ Naturalis Biodiversity Centerpostbus 9517Leiden2300 RAthe Netherlands; ^2^ Institute of Environmental Sciences (CML)University of Leidenpostbus 9500Leiden2300 RAthe Netherlands; ^3^ Institute for Biodiversity and Ecosystem Dynamics (IBED)Computational Geo‐EcologyUniversity of AmsterdamScience Park 904Amsterdam1098 HXthe Netherlands; ^4^ Department of Life SciencesImperial College LondonLondonSW7 2AZUK; ^5^ Royal Botanic Gardens, KewRichmondSurreyTW9 3DSUK

**Keywords:** arbuscular mycorrhizal (AM) fungi, habitat filtering, mycoheterotrophy, phylogenetic niche conservatism, specificity, *Thismia*

## Abstract

- In general, plants and arbuscular mycorrhizal (AM) fungi exchange photosynthetically fixed carbon for soil nutrients, but occasionally nonphotosynthetic plants obtain carbon from AM fungi. The interactions of these mycoheterotrophic plants with AM fungi are suggested to be more specialized than those of green plants, although direct comparisons are lacking.
- We investigated the mycorrhizal interactions of both green and mycoheterotrophic plants. We used next‐generation DNA sequencing to compare the AM communities from roots of five closely related mycoheterotrophic species of *Thismia* (Thismiaceae), roots of surrounding green plants, and soil, sampled over the entire temperate distribution of *Thismia* in Australia and New Zealand.
- We observed that the fungal communities of mycoheterotrophic and green plants are phylogenetically more similar within than between these groups of plants, suggesting a specific association pattern according to plant trophic mode. Moreover, mycoheterotrophic plants follow a more restricted association with their fungal partners in terms of phylogenetic diversity when compared with green plants, targeting more clustered lineages of fungi, independent of geographic origin.
- Our findings demonstrate that these mycoheterotrophic plants target more narrow lineages of fungi than green plants, despite the larger fungal pool available in the soil, and thus they are more specialized towards mycorrhizal fungi than autotrophic plants.

## Introduction

The interaction between arbuscular mycorrhizal (AM) fungi and over 80% of land plants is one of the most widespread mutualisms on Earth (Smith & Read, 2008). AM fungi, which are abundant in most terrestrial ecosystems, are obligatorily associated with the roots of plants and act like extensions of plant root systems to increase the uptake of nutrients, especially phosphorus (Karandashov & Bucher, 2005). However, despite the ubiquity of the interaction, the mechanisms that control its above‐ and belowground diversity are not well understood (van der Heijden *et al*., 2015).

Plant diversity and productivity are significantly influenced by the AM fungal diversity in the soil (van der Heijden *et al*., 1998; Vogelsang *et al*., 2006). A key component of plant productivity is photosynthetic fixation of inorganic carbon. It is this carbon that plants transfer to their mycorrhizal partners in exchange for soil nutrients (Smith & Read, 2008). Occasionally, plant lineages lose the ability to perform photosynthesis but maintain belowground links with mycorrhizal fungi. This phenomenon has long fascinated researchers (e.g. Ramsbottom, 1929; McLennan, 1958) because, in such systems, the expected outcome is that the fungi would also withdraw their participation in the interaction (Sachs & Simms, 2006). Instead, these nonphotosynthetic plants, known as mycoheterotrophs, still harbor AM fungi growing in their roots (e.g. Leake, 1994; Bidartondo *et al*., 2002; Merckx *et al*., 2012).

Mycoheterotrophy is a trophic strategy present in > 20 000 plant species (Merckx, 2013). It is characterized by the absence of photosynthesis, with plants obtaining carbon via the mycorrhizal fungi associated with their roots. The only known way in which AM fungi obtain their carbon is through symbiosis with a photosynthetic plant. Thus, mycoheterotrophic plants must rely on established mutualisms between photosynthetic plants and AM fungi, becoming cheaters within three‐partite interactions (Bidartondo, 2005; Sachs & Simms, 2006). Mycoheterotrophy can occur (1) throughout the life cycle of a plant, such as in some orchids and monotropes, (2) simultaneously with autotrophy, this being termed partial mycoheterotrophy, as in some orchids (Gebauer & Meyer, 2003), or (3) during a short period in the life cycle of a plant, being subsequently replaced by an autotrophic mode of nutrition, such as in many ferns and lycopods, and some orchids (but see Gebauer *et al*., 2016). Thus, mycoheterotrophy can be seen as a dynamic interaction along a continuum of possible outcomes. Because mycorrhizal associations are generally mutualistic (Smith & Read, 2008), it is intriguing why, and which, fungi are part of a mycoheterotrophic interaction. In particular, the differences between mycorrhizal associations of mycoheterotrophic and green plants, and potential preferences for particular fungal lineages, remain poorly understood. Many mycoheterotrophic plants are known to have more specialized interactions with basidiomycete fungi (i.e. they interact with fewer fungal lineages) than ectomycorrhizal green plants, presumably to increase their fitness by optimizing host adaptation (Cullings *et al*., 1996; Bidartondo, 2005). However, the level of mycorrhizal specificity for arbuscular mycoheterotrophic plants remains poorly understood, as comprehensive direct comparisons between AM interactions of mycoheterotrophic and green plants have not been reported. To investigate this, data on the mycorrhizal partners of mycoheterotrophic plants need to be generated and compared with those for the fungal communities associated with green plants.

In the past few years, the study of fungal diversity patterns has become more important in understanding the mechanisms driving plant biodiversity (Öpik *et al*., 2009; Davison *et al*., 2011; Peay *et al*., 2013). Next‐generation sequencing techniques to identify AM fungi allow assessments of the complex fungal communities in soil and plant roots (Toju *et al*., 2014). However, species delimitation of the ancient and apparently strictly asexual AM fungi has long been debated and no consensus has been achieved for suitable molecular markers with sufficient resolution for species‐level identification, nor for the cut‐off values to be used in clustering operational taxonomic units for species prediction (Bruns & Taylor, 2016). Thus, measuring species richness with standard methods may introduce a bias in the assessment of the composition of fungal communities. To better understand how communities are structured, an integration of phylogenetic structure, trait information and community composition can offer relevant insights into the evolutionary and ecological processes shaping communities (Webb *et al*., 2002). At the community scale, species should be segregated based on relative strengths of habitat filtering and competition among similar species. Community structure can be phylogenetically clustered, random, or overdispersed on the phylogeny of the entire available pool of species. For example, Kembel & Hubbell (2006) showed that phylogenetic structure of rainforest tree communities varied among habitats in Panama. They found communities with more closely related taxa than expected by chance (phylogenetically clustered), suggesting strong habitat filtering as the driving force of community assemblages, while other communities were composed of more distantly related taxa (overdispersion), suggesting current or past competitive exclusion between closely related taxa, or convergent evolution of important traits for persistence in such habitats.

In this study, we considered a community to be composed of fungal operational taxonomic units (OTUs) belonging to the same trophic level and the same guild (AM fungi: mycorrhizal fungi from the Glomeromycota phylum) co‐occurring spatially in the roots of a plant. We compared the phylogenetic structure of the fungal communities associated with *Thismia* plants and co‐occurring green plants (comparing plant nutrition types: mycoheterotrophic and autotrophic) confined to the distribution area of the selected mycoheterotrophic lineage, by studying the fungal community composition in their roots using high‐throughput DNA sequencing methods. We considered the level of phylogenetic clustering as a proxy for the mycorrhizal specificity of a plant. A plant species can have specialized mycorrhizal interactions by targeting a single or a few phylogenetically narrow fungal clades, or generalist mycorrhizal interactions by targeting more dispersed phylogenetic fungal lineages. We focused on temperate mycoheterotrophic *Thismia* species to evaluate the mycorrhizal association patterns within a lineage of closely related mycoheterotrophic plants. Because specificity in biotic interactions may differ considerably over a species’ distribution range (Thompson, 2005), we studied the interactions over the geographic range of this *Thismia* clade. Soil samples were included to estimate the fungal pool available for these species. To evaluate general differences in fungal community structure between mycoheterotrophic and autotrophic plants, we used phylogenetic measures to infer community structure.

## Materials and Methods

### Sampling

We sampled temperate forest sites in Australia and New Zealand over the known distribution range of the genus *Thismia* in the region. We visited sites where *Thismia* species are known to occur, and we surveyed other potential occurrence sites with similar habitats (Merckx & Wapstra, 2013). These plants have always been considered extremely rare, and therefore the number of specimens available per site was limited. At each site, one to five *Thismia* specimens were sampled, at least 1 m from each other. This resulted in sampling 18 sites within three broad areas: four in New South Wales (NSW), 10 in Tasmania (TAS) and four in New Zealand (NZ). See Supporting Information Fig. S1 for details.

For each specimen, the entire root system of *Thismia* and the root tips (*c*. 1 cm) of surrounding plants were taken and preserved in 2X CTAB (hexadecyl trimethyl‐ammonium bromide) buffer. The sampling of the surrounding green plants was carried out by selecting up to eight root tips of green plants found in the same soil clump (10 × 10 cm) as *Thismia*. To estimate the fungal pool available for all plant species, soil was sampled from the soil clump as well. Soil was dried on silica gel before DNA extraction. The sampling effort resulted in 99 samples, including mycoheterotrophic plants, green plants and soil (Table S1). All plant roots were identified using molecular methods (Methods S1).

### Assessment of fungal communities using Ion Torrent

Fungal DNA was extracted from the CTAB‐preserved roots with the KingFisher Flex Magnetic Particle Processor (Thermo Fisher Scientific, Waltham, MA, USA) using the NucleoMag 96 Plant Kit (Macherey‐Nagel Gmbh & Co., Düren, Germany). Subsequently, amplicon libraries were created to amplify the internal transcribed spacer (ITS2), using the fungal‐specific primer fITS7 (Ihrmark *et al*., 2012) and ITS4 (White *et al*., 1990) with a unique multiplex identifier (MID) label per sample, following the protocol described in Ihrmark *et al*. (2012). Sequencing was performed with a Personal Genome Machine (Ion Torrent; Life Technologies, Guilford, CT, USA) with 850 flows. Sequences obtained were processed using the uparse algorithm (Edgar, 2013) incorporated in usearch v.7 (http://www.drive5.com/usearch/). Fastq files were screened for quality control and trimmed at the first base with a Phred score of *Q* < 20. Dereplication was performed, singletons and sequences with < 100 bp were filtered out, and the resulting sequences were clustered into OTUs at 97% similarity (Blaalid *et al*., 2013). The taxonomy was assigned to the OTUs with uparse, based on the UNITE + INSD database (10.09.2014) implemented with the current Index Fungorum identification. Only OTUs belonging to the Glomeromycota were kept for further analysis. The raw data were deposited in the National Center for Biotechnology Information (NCBI) Sequence Read Archive under the accession SRP083901. Because of the imbalanced number of specimens obtained for mycoheterotrophic and green plants, we calculated the species richness estimate Chao2 (Chiu *et al*., 2014) for each plant group, using the function specpool in the vegan R package (Oksanen *et al*., 2015).

### Fungal community dissimilarities among samples

We calculated the phylogenetic relatedness between the OTUs to measure community differences between samples. An alignment of the OTU sequences and several reference Glomeromycota taxa from Krüger *et al*. (2011) was constructed with Mafft (Katoh, 2013). Phylogenetic inference on the alignment was performed with Raxmlhpc‐sse3 (Stamatakis, 2014) using the GTR + G + I model of substitution as determined by jmodeltest v.2.1.5 using the Akaike information criterion (AIC) (Darriba *et al*., 2012). The phylogenetic distances among fungal OTUs given the highest likelihood tree were used to obtain a fungal community dissimilarity matrix between all the pairs of samples, using the function Comdist in the picante R package (R Development Core Team, 2008; Kembel *et al*., 2010). This algorithm finds for each fungal OTU in one sample the average distance to all the OTUs in the other sample, and calculates the mean of these phylogenetic distances. The fungal community dissimilarities were visualized by performing a metaMDS in the vegan R package (Oksanen *et al*., 2015). We investigated whether these fungal community dissimilarities differed between the ‘type’ of material (mycoheterotrophic plants, green plants, and soil) and ‘region’ (New South Wales, Tasmania and New Zealand) with a permutational MANOVA using the function adonis in the vegan R package (Oksanen *et al*., 2015).

In addition, we explored whether the community dissimilarity patterns observed in the *Thismia* species were correlated with the plant evolutionary relationships by computing the Mantel test correlation between the fungal community dissimilarity matrix and the phylogenetic distance matrix among the *Thismia* species (see Methods S2 for detailed methods).

### Fungal phylogenetic community structure

To investigate the fungal community structure, we calculated the phylogenetic community structure indices developed by Webb (2000) for community assessment of rainforest trees, which have previously been successfully applied in fungal community studies (e.g. Peay *et al*., 2010; Maherali & Klironomos, 2012). The net relatedness index (NRI) and the nearest taxa index (NTI) measure the degree of phylogenetic clustering of a group of taxa over the whole pool of taxa in a phylogenetic tree or within particular terminal clades, respectively. Positive values indicate that the fungal OTUs are more closely related to one another than expected by chance (phylogenetic clustering), and negative values indicate that the fungal OTUs are more distantly related (phylogenetic evenness). The NRI measures the overall clustering across the phylogeny using the average pairwise distance of all taxa from a community. NRI is then equal to 1 – (MPD_observed_ – MPD_random_)/SD(MPD_random_), where MPD is the mean phylogenetic distance, which measures phylogenetic distance among taxa using the pairwise branch length distances. The pairwise phylogenetic distances among the fungal taxa were obtained from the fungal OTU phylogeny. Numerically, NRI is the inverse of the standardized effect size of the MPD, which compares the average phylogenetic relatedness in the observed and null communities, under a null model of randomizations, standardized by the standard deviation (SD) of phylogenetic distances in the null community (Webb *et al*., 2002). We obtained 999 randomizations, shuffling the tips of the phylogeny from the total pool of fungal taxa. The NTI measures the terminal clustering among the taxa from the community. NTI is then equal to 1 – (MNTD_observed_ – MNTD_random_)/SD(MNTD_random_), where MNTD is the mean nearest phylogenetic taxon distance, which measures the minimal distance separating each species in the community. Numerically, NTI is the inverse of the standardized effect size of the MNTD, calculated similarly to MPD (Webb *et al*., 2002). The standardized effects of the MPD and MNTD measures were calculated using the picante R package (Kembel *et al*., 2010).

In addition, for *Thismia* we reconstructed the NRI value of the most recent common ancestor of the clade based on the plant phylogenetic tree (pruned to contain only one taxon per species) and NRI values per species. Reconstruction was performed using phylogenetic independent constrasts (Felsenstein, 1985) as implemented in ape (Paradis *et al*., 2004).

### General patterns of fungal community structure

Because we were interested in general patterns of community structure, such as the specificity of interactions per trophic strategy, we focused on the NRI for an overall view of community clustering along the phylogeny. The observation of an overall phylogenetic clustered pattern indicates more specialized interactions, where the targeted fungal OTU taxa are more closely related than expected by chance. An overall phylogenetic overdispersion pattern suggests that the interactions are more generalist, where the targeted taxa are more spread out over the phylogenetic tree than expected by chance. In order to test the effects of the ‘type’ of material (mycoheterotrophic, green plants, and soil) on the NRI, we constructed a linear mixed‐effects model with NRI as the response variable and ‘type’ of material as the predictor variable. We considered ‘region’ as a random factor to account for the nonindependence of the collections within and across regions. We then used a post hoc pairwise comparison test (Tukey's honest significant difference (HSD)) to assess whether the three types of material differed significantly from each other in their NRI.

## Results

### Plant identification

We successfully obtained sequences from the roots of 60 specimens of five *Thismia* species, 24 specimens of 11 green plant species and 25 soil samples (see Table S1 for details). The *Thismia* species were identified as *Thismia clavarioides* K. R. Thiele, *Thismia hillii* (Cheeseman) N. Pfeiff., *Thismia megalongensis* C. Hunt, G. Steenbeeke & V. Merckx, *Thismia rodwayi* F. Muell., and a fifth species that remains to be described, here termed *Thismia* sp. For the green plants, we identified the following species (Methods S1): Apocynaceae sp.; *Laurelia novae‐zelandiae* A. Cunn., and *Doryphora sassafras* Endl. (Atherospermataceae); *Bignoniaceae* sp.; *Ceratopetalum apetalum* D. Don (Cunoniaceae); *Beyeria viscosa* Labill. (Euphorbiaceae); *Acacia* sp. (Fabaceae); *Beilschmiedia tawa* (A. Cunn.) Kirk (Lauraceae); *Pomaderris apetala* Labill. (Rhamnaceae); *Nematolepis* sp. Turcz. (Rutaceae); and Vitaceae sp. (Table S1).

The success rate of sequencing Glomeromycota fungi from the autotrophic plants was considerably lower than for *Thismia*, and for several surrounding root samples we failed to obtain Glomeromycota OTUs. Some of the autotrophic plants are putatively ectomycorrhizal, which may explain the absence of Glomeromycota OTUs in surrounding roots. *Pomaderris apetala* and *Acacia* sp. can be both ectomycorrhizal and AM, and all other species are described as AM (Brundrett, 2008), except for *Beyeria viscosa* and *Nematolepis* sp. for which the mycorrhizal status is unknown, making them suitable for the comparisons in the downstream analysis.

### Fungal sequences

**Figure 1:**
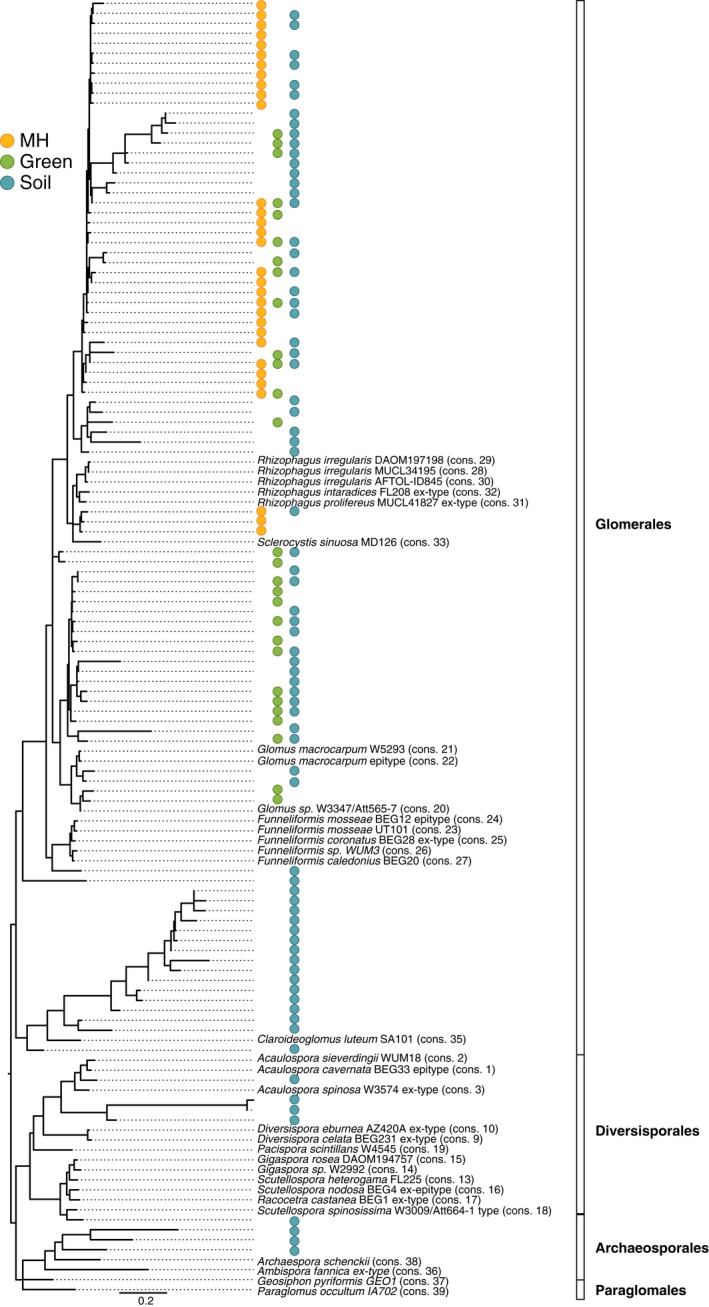
Highest likelihood tree (LnL = −10519.28) showing the phylogenetic relationships among the Glomeromycota operational taxonomic units (OTUs) found in all the samples, including several reference sequences. The colored circles indicate the presence of the fungal OTUs according to plant group (mycoheterotrophic, yellow; autotrophic, green) and the pool of fungal OTUs present in the soil (blue). Mycoheterotrophic plants of the genus *Thismia* are associated with fungi in the Glomerales family (one subclade: *Rhizophagus/Sclerocystis* sp.); and green plants are also associated with fungi in the Glomerales family (two subclades: *Rhizophagus/Sclerocystis* sp. and *Glomus* sp.). The soil also harbors fungi from the Glomerales family, and also from the Diversisporales and Archaeosporales families within the Glomeromycota phylum.

Ion Torrent sequencing produced 4038 169 raw sequences, of which 3836 916 passed the quality filtering. After the quality control steps, the resulting sequences were clustered at 97% similarity, generating 466 OTUs, of which 99 OTUs were assigned to Glomeromycota and kept for subsequent analysis. Of these, 31 OTUs were found in the mycoheterotrophic plants, 28 OTUs were found in the green plants and 69 OTUs were found in the soil. The number of OTUs was not linearly correlated to the variable number of reads per sample, and thus neither is it linearly correlated to the number of OTUs per type of material (see Fig. S2). Using the Chao2 estimator, we obtained richness estimates of 32.26 ± SD 1.77 for mycoheterotrophic plants, 36.61 ± SD 6.30 for green plants, and 101.67 ± SD 15.12 for soil. Fig. 1 shows the highest likelihood phylogeny among the fungal OTUs and respective presence in mycoheterotrophic plants, green plants and soil. The fungal communities of the five *Thismia* species included Glomeromycota in the *Rhizophagus*/*Sclerocystis* sp. subclade; for the green plants, the same clades of fungi were present with the addition of the *Glomus* sp. subclade; in the soil, Glomerales, Diversisporales and Archaeosporales fungi were present.

### Fungal community dissimilarities

**Figure 2:**
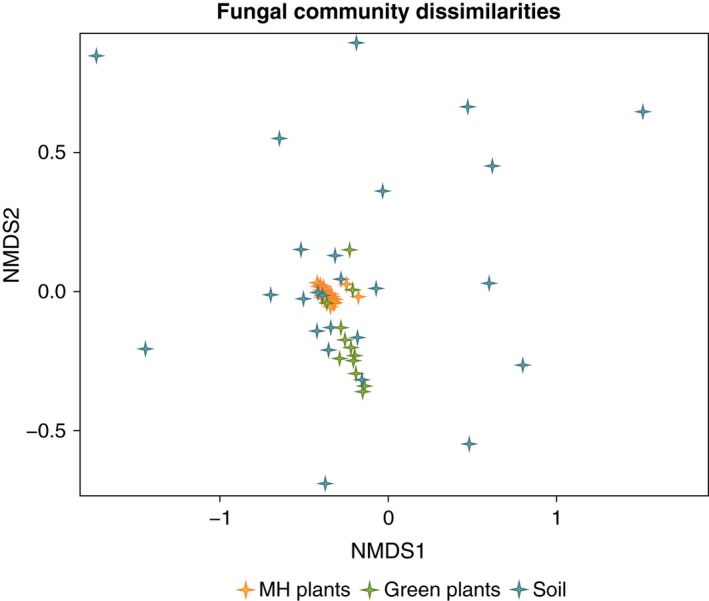
Nonmetric multidimensional scaling plot (*metaMDS*) showing an ordination of the fungal community dissimilarities (*Comdist*) among all the samples. The fungal community dissimilarities are calculated based on the average phylogenetic distance between each fungal operational taxonomic unit (OUT) in one sample and the total OTUs in the other sample. Each symbol represents the *Comdist* of the fungal communities including all the OTUs found in each species per site. Permutational MANOVA (*Adonis*) showed significant fungal community dissimilarity between mycoheterotrophic (MH) *Thismia* plants, green plants, and soil (*F *=* *25.4; *R* ^2^ = 0.486; *P *=* *0.001).

Fungal community dissimilarities were calculated among all the samples, including mycoheterotrophic plants, green plants and soil. In Fig. 2, a nonmetric multidimensional scaling plot shows an ordination of the fungal community dissimilarities. Furthermore, we found no phylogenetic signal on the fungal community dissimilarities among the different *Thismia* species (Mantel test: *r *=* *0.092; *P *=* *0.196). Thus, we proceeded with the fungal community dissimilarity analysis including the green plants and looked for patterns within the ‘type’ of material (mycoheterotrophic plants, green plants and soil), and we also looked for geographic patterns (‘region’: Tasmania, New South Wales and New Zealand). Permutational MANOVA (*Adonis*) showed significant fungal community dissimilarity for ‘type’ of material (*F *=* *25.4; *R* ^2^ = 0.486; *P *=* *0.001), but not for ‘region’ (*F *=* *0.925; *R* ^2^ = 0.018; *P *=* *0.427). These results suggest a distinctive and specific association pattern of the fungal communities for mycoheterotrophic plants, green plants and soil, regardless of the region in which they occur.

### Fungal phylogenetic community structure

We observed that all the mycoheterotrophic plants exhibited positive and significant NRI and NTI values (Table 1), which indicates a significant phylogenetic structure of the fungal communities. The two indices were correlated (Fig. S3). By contrast, most of the green plants and soil communities were phylogenetically randomly structured for both indices (Table 1). The roots of mycoheterotrophic plants tended to be colonized by AM fungi that were more closely related than expected by chance. The green plants tended to show no clear pattern in general, except for five species that presented phylogenetic structure. The soil also seemed to be mostly randomly phylogenetically structured. Overall, the two indices were concordant.

**Table 1:** Net relatedness index (NRI) and nearest taxa index (NTI) results for the fungal communities of mycoheterotrophic (MH) plants (*Thismia*), green plants and soil

Type	Samples	*n*	NRI	RGR	NTI	RGR
MH plants	*T. rodwayi* 1 TAS	12	4.14^b^	999	2.38^b^	999
	*T. rodwayi* 2 TAS	4	2.16^b^	996	1.77^b^	996
	*T. rodwayi* 3 TAS	9	3.42^b^	999	2.13^b^	999
	*T. rodwayi* 4 TAS	14	4.49^b^	999	2.44^b^	999
	*T. rodwayi* 5 TAS	12	4.13^b^	999	2.34^b^	999
	*T. rodwayi* 6 TAS	3	1.79^b^	999	1.65^b^	998
	*T. rodwayi* 7 TAS	8	3.23^b^	999	2.12^b^	999
	*T. rodwayi* 8 TAS	9	3.41^b^	999	2.14^b^	999
	*T. rodwayi* 9 TAS	12	4.20^b^	999	2.41^b^	999
	*T. rodwayi* 10 TAS	8	3.24^b^	999	2.09^b^	999
	*T. clavarioides* NSW	5	2.53^b^	999	1.72^b^	995
	*Thismia* sp. NSW	3	1.80^b^	997	1.55^b^	998
	*Thismia* sp. NSW	4	1.74^b^	979	1.44^b^	957
	*T. hillii* NSW	7	3.00^b^	999	2.08^b^	999
	*T. megalongensis* NSW	6	2.67^b^	999	2.08^b^	999
	*T. hillii* 1 NZ	9	3.29^b^	999	2.17^b^	999
	*T. hillii* 2 NZ	4	1.95^b^	991	1.65^b^	994
	*T. hillii* 3 NZ	6	2.68^b^	998	2.04^b^	999
	*T. hillii* 4 NZ	6	2.61^b^	999	1.96^b^	999
Green plants	*Acacia* sp. TAS	2	0.92	818	0.92	808
	*Beyeria viscosa* TAS	2	0.53	567	0.54	545
	*Pomaderris apetala* TAS	4	1.13	788	1.11	840
	*Nematolepis* sp. TAS	2	1.24^a^	919	1.21^a^	935
	*Acacia* sp. NSW	2	1.20	892	1.19	868
	Bignoniaceae sp. NSW	3	1.66^b^	986	1.52^b^	975
	*Ceratopetalum apetallum* NSW	3	1.02	774	0.97	788
	*Doryphora sassafras* NSW	10	2.97^b^	999	1.70^b^	975
	Vitaceae sp. NSW	7	2.15^b^	988	1.44^a^	921
	Apocynaceae sp. NSW	6	1.30	887	0.84	739
	*Beilschmiedia tawa* NZ	3	0.75	656	0.55	624
	*Laurelia novae‐zelandiae* NZ	13	2.28^b^	983	1.93^b^	993
Soil	Soil 1 TAS	9	0.06	523	−0.91	181
	Soil 2 TAS	2	1.19	890	1.19	892
	Soil 3 TAS	2	−1.13	153	−1.14	157
	Soil 4 TAS	6	2.65^b^	999	2.07^b^	999
	Soil 5 TAS	11	−1.26	105	−1.85	38
	Soil 6 TAS	5	0.59	735	0.46	669
	Soil 7 TAS	2	−1.43	69	−1.46	67
	Soil 8 TAS	3	0.74	679	0.84	777
	Soil 9 TAS	6	1.16	829	0.67	722
	Soil 10 TAS	9	−1.86	30	2.02^b^	994
	Soil 11 TAS	2	1.32^b^	993	1.39^b^	995
	Soil 12 TAS	13	−0.26	385	1.86^b^	982
	Soil 13 TAS	14	−0.92	184	1.04	855
	Soil 14 TAS	2	−1.49	70	−1.52	71
	Soil 15 TAS	7	0.65	717	0.61	709
	Soil 16 TAS	4	2.20^b^	996	1.77^b^	997
	Soil 17 TAS	5	0.79	757	0.57	717
	Soil 18 TAS	3	0.04	561	0.46	648
	Soil 1 NSW	4	−0.67	273	−0.44	312
	Soil 2 NSW	16	−0.38	376	2.36^b^	998
	Soil 3 NSW	3	1.34^a^	933	1.36^a^	945
	Soil 4 NSW	3	1.65^b^	971	1.50^b^	993
	Soil 5 NSW	2	0.33	496	0.30	474
	Soil 6 NSW	3	0.80	667	0.95	812
	Soil NZ	6	1.30	864	1.29	893

Samples, species per site; *n*, number of OTUs in a community; RGR, number of times the observed NRI or NTI was greater than the value obtained for the random permuted communities.

a Communities significantly structured at the *P *=* *0.10 level.

b Communities significantly structured at the *P *=* *0.05 level.

The NRI of the most recent common ancestor of the *Thismia* clade was reconstructed to be 4.00 (95% confidence interval (CI) 3.26–4.74; see Fig. S4).

### General patterns of fungal community structure

**Figure 3:**
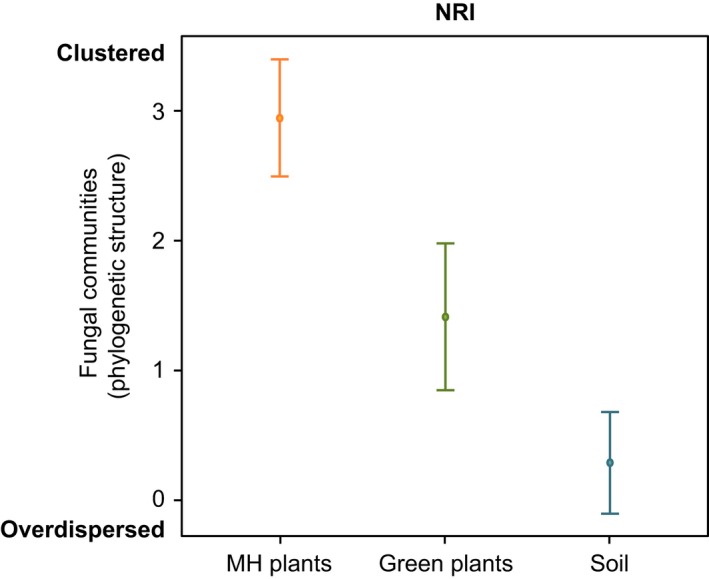
Fungal community structure based on the net relatedness index (NRI) for each species per site. The graph represents the fungal communities’ phylogenetic dispersion patterns as explained by the ‘type’ of material (mycoheterotrophic (MH) plants, green plants and soil). Negative NRI values indicate that the fungal communities are overdispersed in the phylogenetic tree, while positive NRI values indicate phylogenetic clustering. The NRI was significantly different in MH plants compared with green plants and soil. MH plants harbor more phylogenetically clustered AM fungal communities in their roots than green plants and the soil. Green plants also have significantly more clustered fungal communities than the soil. The mixed‐effects model estimates with 95% confidence intervals are shown. See Supporting Information Table S2 for statistical details.

The mixed‐effects model results showed that the fungal community structure was significantly explained by the ‘type’ of material. The fungal communities associated with the mycoheterotrophic plants were significantly more closely related to each other than in the case of the green plants and the soil. Likewise, for the green plants, the fungal communities were also significantly more closely related to each other than in the soil (see Fig. 3; Table S2).

## Discussion

The plant sampling was designed to investigate the fungal community structure of closely related mycoheterotrophic plant species over their entire geographic range and, at the same time, compare their fungal community structure with that of the surrounding autotrophic plants, as a proxy for mycoheterotrophic and autotrophic types of nutrition, respectively. The soil data were used as a proxy for the diversity of local AM fungi. As expected, the soil presented a higher fungal diversity compared with individual plants, as it harbors the fungal reservoir from which the plant species obtain their fungal partners (Table S1).

Our results indicate that, in general, mycoheterotrophic and green plants have distinct fungal community compositions with no geographic pattern (Fig. 2; *Adonis* test). In addition, the five closely related *Thismia* species tended to associate with more closely related AM fungi more often than expected by chance. Observations of other cases of mycoheterotrophic species growing on narrow phylogenetic lineages of AM fungi have been reported previously, for example *Arachnitis* (Bidartondo *et al*., 2002), *Afrothismia* (Merckx & Bidartondo, 2008), *Burmannia* (Ogura‐Tsujita *et al*., 2013) and *Petrosavia* (Yamato *et al*., 2011). Moreover, we observed that the phylogenetic structure of the fungal communities can vary according to the type of nutrition of a plant (i.e. mycoheterotrophic vs autotrophic; see Fig. 3).

For the mycoheterotrophic plants, we detected significant NRI and NTI values (Table 1). These two indices provide information about community structure that is different from that provided by richness or taxonomic composition. In view of the unequal number of specimens of mycoheterotrophic and green plants and differences in sequencing success, we calculated the improved richness estimator Chao2 of Chiu *et al*. (2014), incorporating a small‐sample correction. This estimator reduces the bias when the heterogeneity of species detection probabilities is relatively high (Chiu *et al*., 2014). While the estimated richness was higher for the green plants than for the mycoheterotrophic plants, the observed richness was higher for the mycoheterotrophic plants. Considering phylogenetic relatedness among the taxa, we found that, within the Glomeraceae family, the fungi associated with mycoheterotrophic plants belonged to one subclade, while green plants had fungal partners in two subclades (Fig. 1). Thus, the higher estimated richness for the green plants corresponded to a higher phylogenetic diversity compared with the mycoheterotrophic plants.

The phylogenetic clustering pattern observed in the mycoheterotrophic plants’ fungal communities reflected ecological rather than biogeographic patterns, as there was no geographical structure of the fungal communities. Moreover, the tendency of *Thismia* species to target the same narrow clades of AM fungi (Fig. S5), and their similar levels of mycorrhizal specificity (Table 1), also reconstructed to have been present in the most recent common ancestor of the clade (Fig. S4), strongly suggest that the high level of mycorrhizal specificity is prone to phylogenetic niche conservatism (Harvey & Pagel, 1991; Lord *et al*., 1995), that is, the tendency of these *Thismia* species to retain similar ecological traits (i.e. similar fungal communities) over time (Wiens & Graham, 2005; Wiens *et al*., 2010). The phylogenetic niche conservatism observed in *Thismia* may be attributable to a reduction in the potential range of ecological character evolution caused by fixation of ancestral traits, enabling the descendants within this plant lineage to be more successfully adapted in particular and similar habitat types (Lord *et al*., 1995). The reason for the preference for targeting certain lineages of AM fungi in this mycoheterotrophic interaction is still not well understood. It is certainly not caused by a limited local availability of AM fungi, because we detected a much larger and phylogenetically broader pool of available fungi in the soil. Similar to the explanation for the high host specificity of many parasites, the mycoheterotrophs may fine‐tune their physiology on particular lineages of fungi to maximize their carbon uptake (Leake & Cameron, 2010). Alternatively, the mycoheterotrophic plants may be rejected by most fungal lineages in the pool of available fungi, and therefore the pattern would result from an evolutionary arms race (Bidartondo, 2005). Therefore, it is our interpretation that the fungal communities associated with these mycoheterotrophic plants might have been shaped not only by habitat filtering (occurrence of the fungal partners in space), but also by an effect of the ancestry of the plant species, which allow this local third‐party cheater (*Thismia*) to participate in the globally mutualistic AM interaction with autotrophic plants.

For the green plants, some species showed significantly phylogenetically clustered AM fungal communities (Table 1). Specific patterns in the fungal associations of green plants have been previously reported in other studies (e.g. Öpik *et al*., 2009; Davison *et al*., 2011; Peay *et al*., 2013). Nonetheless, other green plants in our study presented a randomly assembled fungal community. This may reflect a different community structure according to plant species, but it may also be an effect caused by an underrepresentation of the fungal communities, which was more likely to occur in the green plants than in the mycoheterotrophic plants because of sampling method limitations. For the green plants we could only collect a few centimeters of the extensive root system, so, because of the scattered pattern of AM fungal colonization along the roots, we may have assessed a limited fraction of the whole diversity, while for the mycoheterotrophic plants, we collected the entire small root system. Nevertheless, we do not think that this underrepresentation of green plants’ fungal communities introduced bias to our results, because although it could be assumed that we were observing partial diversity, we obtained less phylogenetic clustering in green plants than in mycoheterotrophic plants. The phylogenetic clustering of these communities would become even more diluted with the introduction of more phylogenetically different taxa in the analysis, and therefore the specificity would decrease (Webb, 2000).

Generally, the comparison of fungal communities associated with mycoheterotrophic and autotrophic plants showed that this particular lineage of mycoheterotrophic *Thismia* species have significantly more specialized interactions than the green plants living in the same regions (Fig. 3). Mycoheterotrophic plants had significantly more specialized fungal interactions than green plants, because the mycoheterotrophs showed higher NRI values almost exclusively. Similarly, mycoheterotrophic plants also had generally higher ranks of NTI values (Table 1). This suggests that, within the Glomerales subclade targeted by mycoheterotrophic plants, these plants also tend to target specific lineages at a lower taxonomic level. These results support the view that mycoheterotrophic mycorrhizal interactions are highly specialized. By contrast, green plants did not always show significantly clustered patterns. If we excluded the green plants for which we detected fewer than three OTUs (minimum number of OTUs found in the *Thismia* species), we found that half of the autotrophic plants (*Doryphora sassafras*,* Bignoniaceae* sp., *Laurelia novae‐zelandiae* and Vitaceae sp.) tended to associate with more closely related main lineages of AM fungi than expected by chance, but generally with lower ranks of positive NRI and NTI values compared with *Thismia*. We also found that the other half (Apocynaceae sp., *Ceratopetalum apetalum*,* Beilschmiedia tawa* and *Pomaderris apetala*) did not present a significantly clustered pattern. In conclusion, even though some green plants may also tend to target more closely related AM fungal taxa than expected by chance, in general these green plants have less specialized interactions compared with *Thismia*.

In this study, we tested the association between these two ecological traits (type of plant nutrition (mycoheterotrophic vs autotrophic) and phylogenetic fungal community structure) for these *Thismia* species and surrounding green plants. The study of fungal community structure needs to be extended to other distantly related lineages of mycoheterotrophic plants before we make generalizations about the processes shaping the fungal interactions involved in mycoheterotrophy. Moreover, understanding how the fungal communities associated with plants in general are assembled can provide us with knowledge of how belowground ecological processes influence the global distribution of plants in ecosystems.

## Author contributions

S.I.F.G. and V.S.F.T.M. planned and designed the research, V.S.F.T.M. collected the samples, S.I.F.G. generated the data and performed the analysis, J.A‐G. participated in the data analysis, M.I.B. contributed to the interpretation of the results, and S.I.F.G. wrote the manuscript. All the authors commented on the final version of the paper.

## Supporting information

Please note: Wiley Blackwell are not responsible for the content or functionality of any Supporting Information supplied by the authors. Any queries (other than missing material) should be directed to the *New Phytologist* Central Office.

**Fig. S1** Map of sampling localities.
**Fig. S2** Plot of the total number of OTUs against the total number of reads.
**Fig. S3** Relationship between the net relatedness index and nearest taxa index.
**Fig. S4** Ancestral state reconstruction of the NRI on the species‐level *Thismia* phylogeny.
**Fig. S5** Tanglegram of the interactions between mycoheterotrophic species of *Thismia* and AM fungal OTUs.
**Table S1** Summary of the samples used in the analysis
**Table S2** Statistical results of the mixed‐effects model and multiple comparison analysis explaining the fungal communities’ phylogenetic dispersion patterns by the ‘type’ of material (mycoheterotrophic plants, green plants, and soil), using ‘region’ as a random factor
**Methods S1** Plant identification.
**Methods S2** *Thismia* phylogenetic relationships.
