# Supplementary material for: Arbuscular mycorrhizal interactions of mycoheterotrophic Thismia are more specialized than in autotrophic plants
Source: New Phytol. 2016 Oct 14;213(3):1418–27. doi: 10.1111/nph.14249 (PMC5248637; doi:10.1111/nph.14249)
Supplement: Supplementary file 1 — Fig. S1 Map of sampling localities. Fig. S2 Plot of the total number of OTUs against the total number of reads. Fig. S3 Relationship between the net relatedness index and nearest taxa index. Fig. S4 Ancestral state reconstruction of the NRI on the species‐level Thismia phylogeny. Fig. S5 Tanglegram of the interactions between mycoheterotrophic species of Thismia and AM fungal OTUs. Table S1 Summary of the samples used in the analysis Table S2 Statistical results of the mixed‐effects model and multiple comparison analysis explaining the fungal communities’ phylogenetic dispersion patterns by the ‘type’ of material (mycoheterotrophic plants, green plants, and soil), using ‘region’ as a random factor Methods S1 Plant identification. Methods S2 Thismia phylogenetic relationships. [file NPH-213-1418-s001.pdf]

New Phytologist

New Phytologist Supporting Information

Article title: Arbuscular mycorrhizal interactions of mycoheterotrophic *Thismia* are more specialized than autotrophic plants
Authors: Sofia I. F. Gomes, Jesús Aguirre-Gutiérrez, Martin I. Bidartondo and Vincent S. F. T. Merckx
Article acceptance date: 5 September 2016

The following Supporting Information is available for this article:

Fig. S1 Map of sampling localities.

Fig. S2 Plot of the total number of OTUs against the total number of reads.

Fig. S3 Relationship between the net relatedness index and nearest taxa index.

Fig. S4 Ancestral state reconstruction of the NRI on the species level *Thismia* phylogeny.

Fig. S5 Tanglegram of the interactions between mycoheterotrophic species of *Thismia* and AM fungal OTUs.

Table S1 Summary of the samples used in the analysis.

Table S2 Statistical results of the mixed-effects model and multiple comparison analysis explaining the fungal communities' phylogenetic dispersion patterns by the 'type' of material (MH plants, green plants, soil), using 'region' as a random factor.

Methods S1 Plant identification.

Methods S2 *Thismia* phylogenetic relationships.

New Phytologist

Fig. S1 Map of sampling localities. A total of 18 sites were sampled within three broad areas: 4 in New South Wales, 10 in Tasmania and 4 in New Zealand. The number of *Thismia* specimens sampled per site is represented in parentheses. Inset shows a flower of *Thismia rodwayi* as illustration of one of the species (bar, 1 cm).

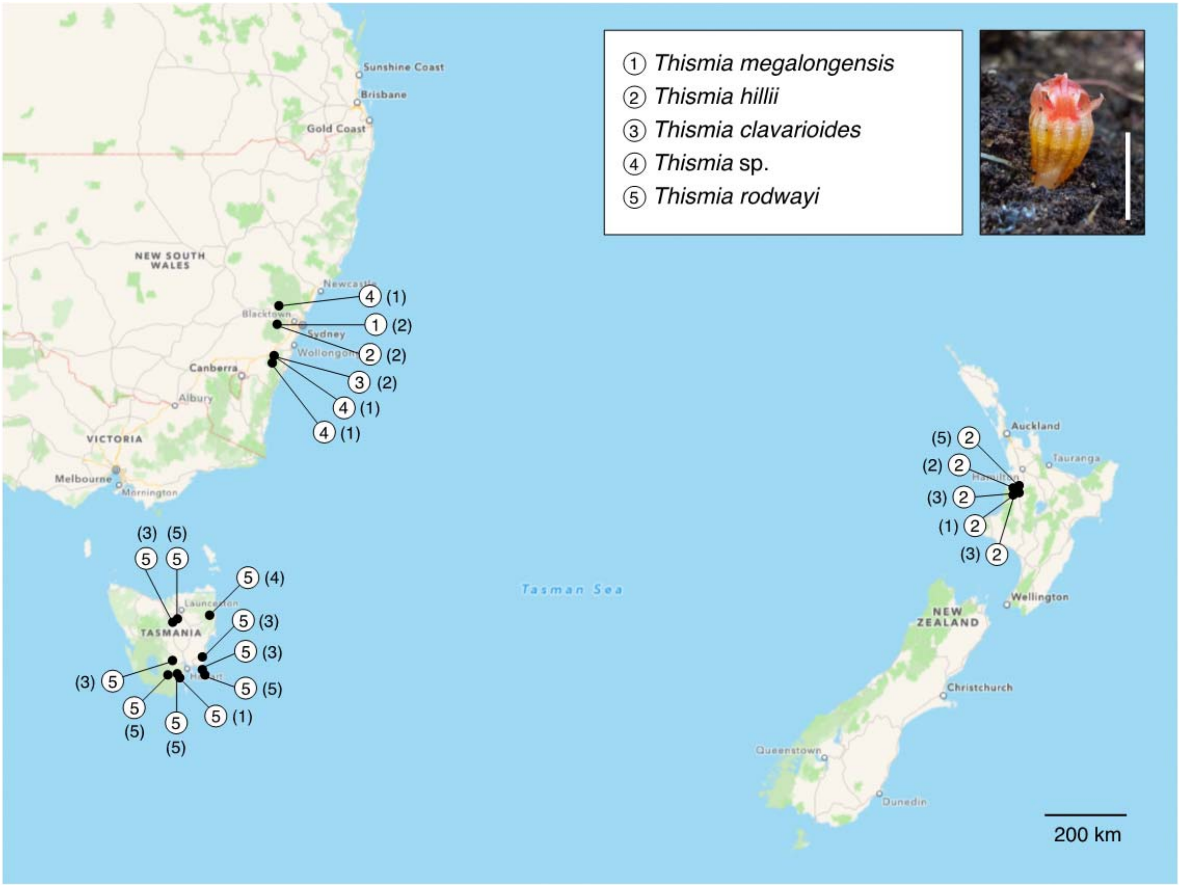

New Phytologist

Fig. S2 Plot of the total number of OTUs against the total number of reads. The total number of reads originated by the Ion Torrent run after the quality control steps (excluding sequences with Q < 20) was plotted against the number of OTUs after clustering at 97% similarity, across all samples. Pearson correlation test (r = 0.31, $P < 0.05$) shows a weak correlation between the number of reads and the number of OTUs generated, but there is no a linear relationship (r^2 = 0.096, $P < 0.05$).

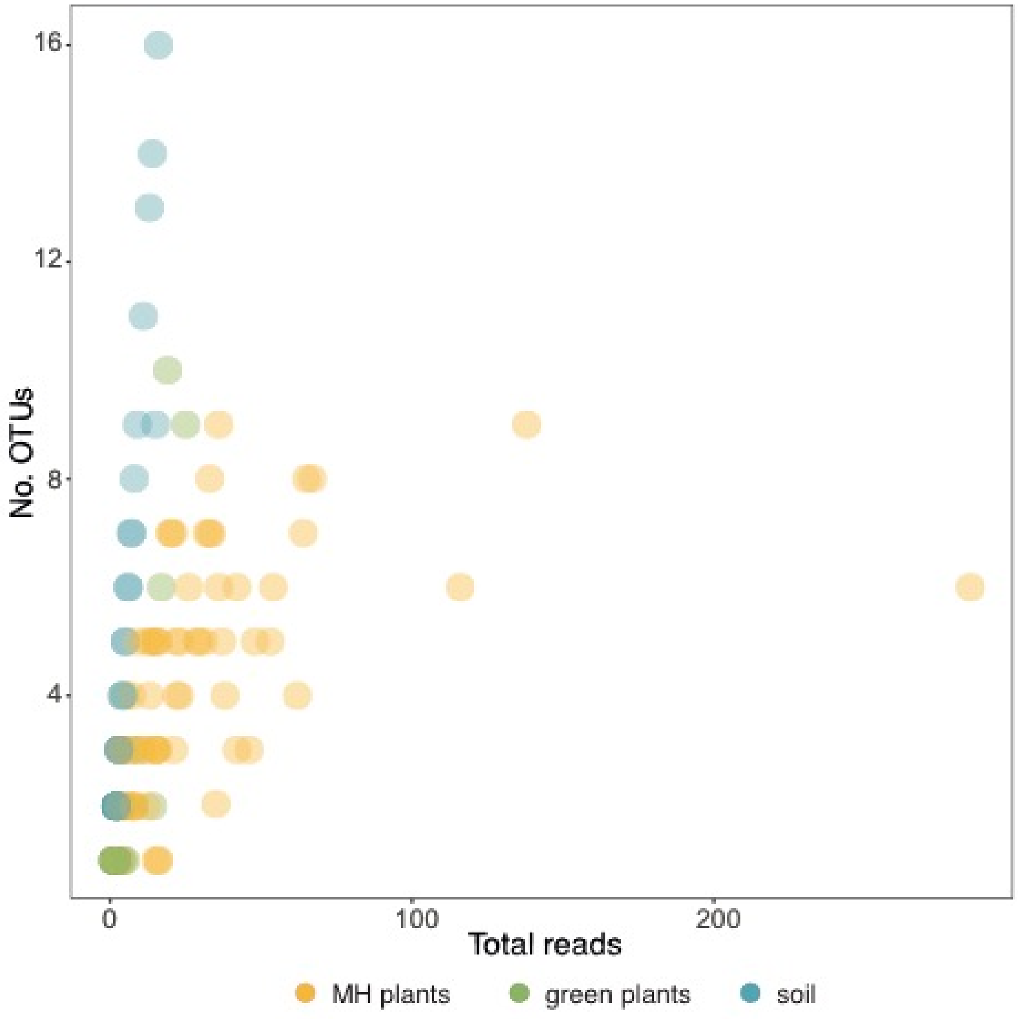

New Phytologist

Fig. S3 Relationship between the net relatedness index and nearest taxa index. Pearson correlation test (r = 0.77, $P < 0.001$) shows that both indices (see Table 1) are correlated, indicating that an overall fungal communities' clustering or dispersal on the deeper nodes of the tree (NRI) corresponds to a similar extent of terminal clustering or dispersal, i.e., near the tips of the tree (NTI).

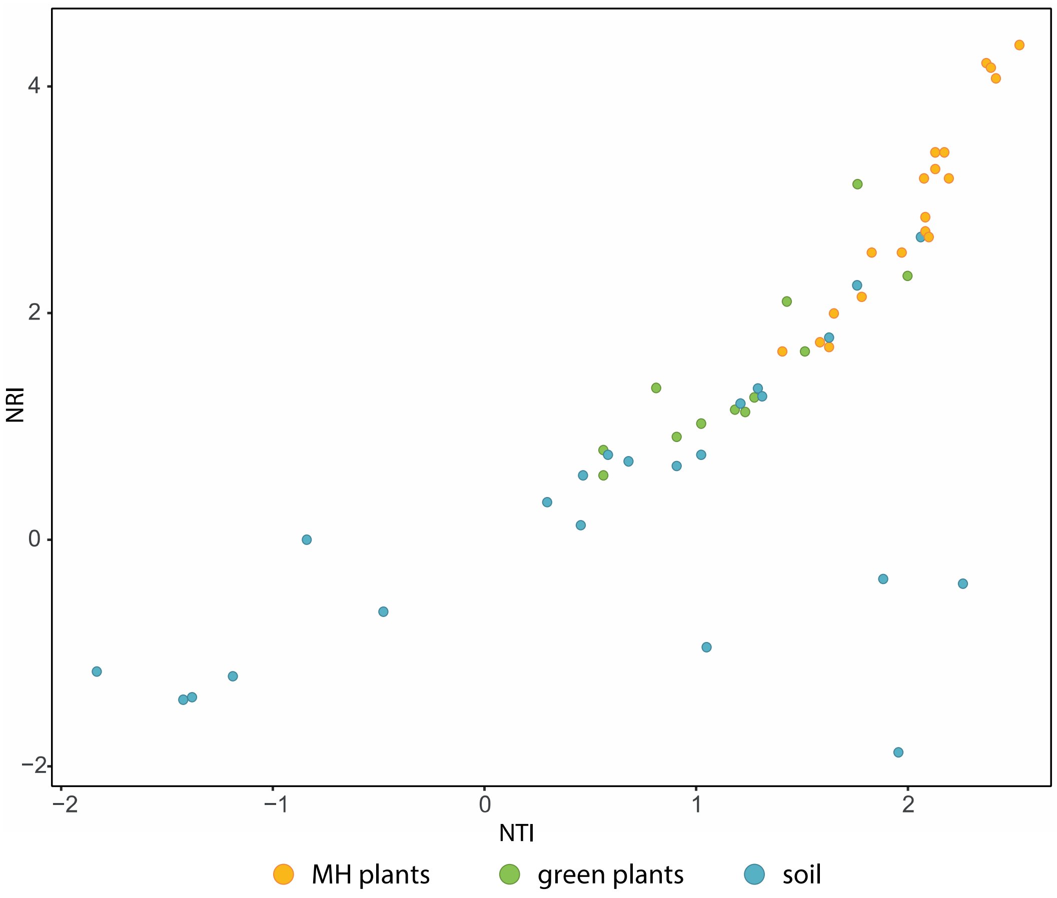

New Phytologist

Fig. S4 Ancestral state reconstruction of the NRI on the species level *Thismia* phylogeny. For each species, the observed NRI is shown at the tips and the reconstructed values are shown on the nodes. The reconstructed NRI of the most common recent ancestor of this lineage (4.00; 95% CI: 3.26–4.74) is within the range of the extant species, which means that the ancestor had similar mycorrhizal specificity, and thus already showed specialized interactions.

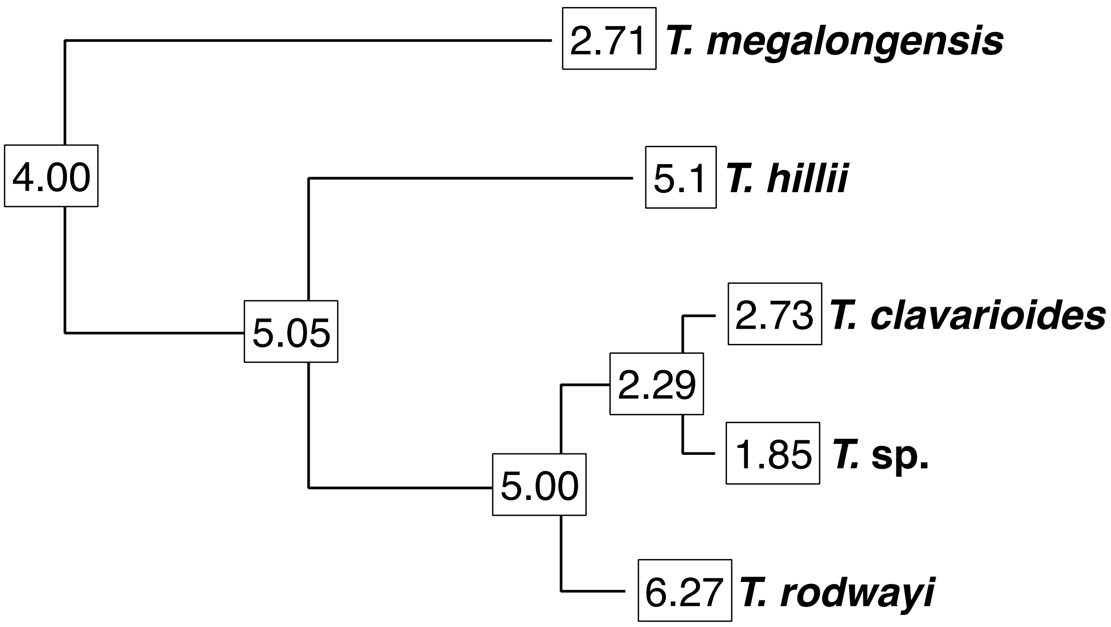

New Phytologist

Fig S5 Tanglegram of the interactions between mycoheterotrophic species of *Thismia* and AM fungal OTUs. The phylogenetic tree of *Thismia* is represented on the left side (see Supporting Information, Methods S2 for details on the phylogenetic relationships of the five species of *Thismia*), and the phylogenetic tree of the AM fungal OTUs on the right side (same as Fig. 1). The tanglegram was built using the *ape* R-package. The figure shows extensive overlap in the fungal interactions within the five *Thimia* species.

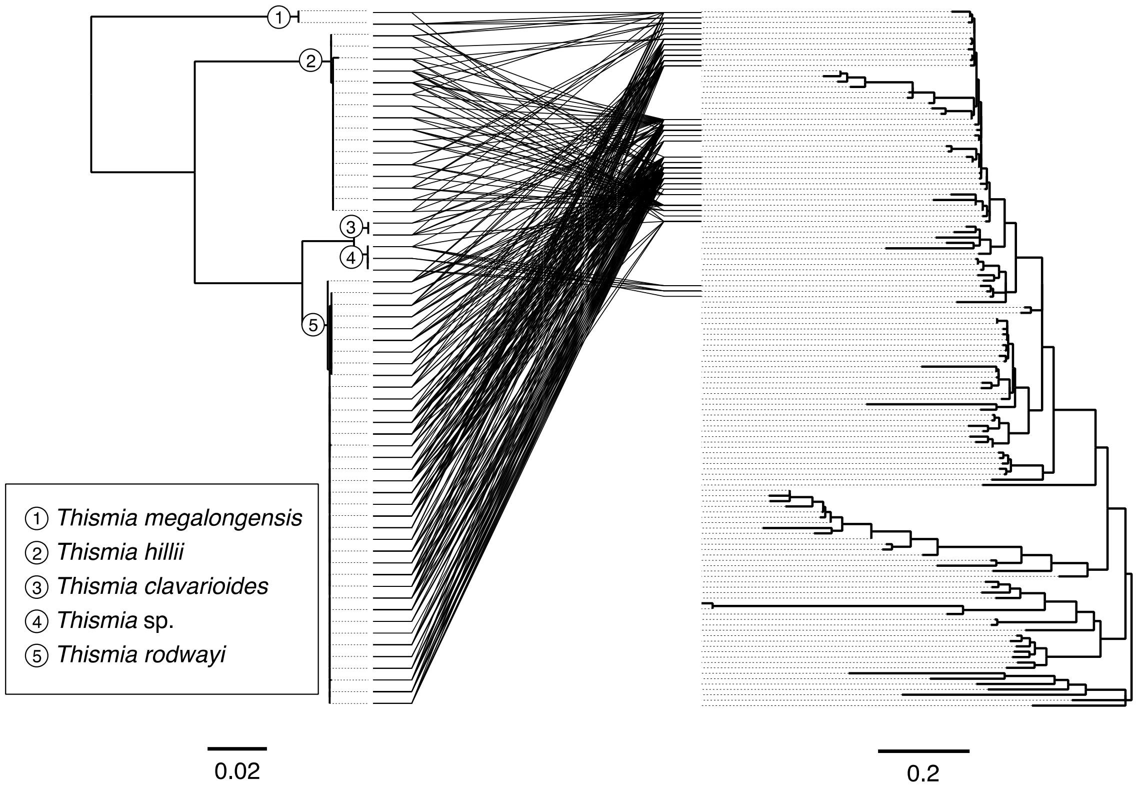

Table S1 Summary of the samples used in the analysis. In total, we found 99 Glomeromycota OTUs in 5 MH *Thismia* species and 11 green plant species. The table shows the number of samples (total 109), which were pooled in 61 samples for Ion Torrent sequencing. 'No. OTUs' corresponds to the number of unique OTUs found per plant species per site, or per locality in the case of soil samples. Species identification of the green plants is showed to the lowest taxonomical level possible to identify based on *matK* or *trnL* genetic markers.

Species	No samples	Pooled samples	Type	Location	No. OTUs
Thismia rodwayi	37	10	MH	Tasmania	23
Thismia megalongensis	2	1	MH	New South Wales	6
Thismia hillii	2	1	MH	New South Wales	7
Thismia clavarioides	2	1	MH	New South Wales	5
Thismia sp.	3	3	MH	New South Wales	5
Thismia hillii	14	5	MH	New Zealand	16
Beyeria viscosa	1	1	green	Tasmania	2
Pomaderris apetala	4	1	green	Tasmania	4
Nematolepsis sp.	1	1	green	Tasmania	2
Acacia sp.	2	1	green	Tasmania	2
Ceratopetalum apetalum	2	1	green	New South Wales	3
Acacia sp.	1	1	green	New South Wales	2
Doryphora sassafras	2	2	green	New South Wales	10
Bignoniaceae sp.	1	1	green	New South Wales	3
Vitaceae sp.	3	2	green	New South Wales	7
Apocynaceae sp.	3	1	green	New South Wales	6
Beilschmiedia tawa	2	1	green	New Zealand	4
Laurelia novae-zelandiae	2	2	green	New Zealand	14
Soil	18	18	soil	Tasmania	56
Soil	6	6	soil	New South Wales	29
Soil	1	1	soil	New Zealand	7

New Phytologist

Table S2 Statistical results of the mixed-effects model and multiple comparison analysis explaining the fungal communities' phylogenetic dispersion patterns by the 'type' of material (MH plants, green plants, soil), using 'region' as a random factor. The multiple linear comparisons test whether the degree of phylogenetic dispersion of the fungal communities is significantly different among mycoheterotrophic plants, green plants and soil.

**Comparisons**	**Coefficient**	**SE**	***P*-value**
MH plants	2.97	0.23	< 0.001
green plants	1.43	0.29	< 0.001
soil	0.30	0.20	0.433
MH plants– green plants	1.54	0.37	< 0.001
MH plants – soil	2.68	0.31	< 0.001
green plants – soil	1.13	0.36	0.007

The last three rows represent the multiple linear comparisons. Adjusted Tukey's *P-values* are reported

New Phytologist

Methods S1 Plant identification

The plants collected in this study consisted of mycoheterotrophic and green species. The mycoheterotrophic species were identified by the genetic markers ITS, using the primers ITS1 and ITS4 (White *et al.,* 1990) and cob, using the primers COB1F and COB1R (Petersen *et al.,* 2006; GenBank accessions KX790794–KX790923). Partial *matK* sequences were obtained from the root tips DNA extractions of the surrounding plants and leaf samples of identified species collected at the sites using primers trnK685F and matK1777R (Hu *et al.,* 2000). For several plant samples from sites in New South Wales this did not result in amplification products. For these plants partial *trnL* sequences were obtained with primers trnL-f and trnL-15 (Taberlet *et al.,* 1991). Root tips were identified based on their sequence similarity with the leaf samples and/or BLAST searches on GenBank.

References

Hu J-M, Lavin M, Wojciechowski MF, Sanderson MJ. 2000. Phylogenetic systematics of the tribe Millettieae (Leguminosae) based on chloroplast *trnK/matK* sequences and its implications for evolutionary patterns in Papilionoideae. *American Journal of Botany* **87:** 418–430.

Taberlet P, Gielly L, Pautou G, Bouvet J. 1991. Universal primers for amplification of three non-coding regions of chloroplast DNA. *Plant Molecular Biology* **17:** 1105–1109.

Petersen G, Seberg O, Davis JI, Stevenson DW. 2006. RNA editing and phylogenetic reconstruction in two monocot mitochondrial genes. *Taxon* **55**: 871–886.

White TJ, Bruns T, Taylor JSL. 1990. Amplification and direct sequencing of fungal ribosomal RNA genes for phylogenetics. In: Innis M, Gelfand D, Sininsky J, White T, eds. *PCR protocols: a guide to methods and applications*. San Diego, CA, USA: Academic Press, 315–332.

Methods S2 *Thismia* phylogenetic relationships. Nuclear ITS and mitochondrial *cob* sequences were used to investigate the relationships at population level among all sampled *Thismia* specimens. Alignments were produced with MAFFT (Katoh*,* 2013). Phylogenetic inference on the aligned datasets (ITS, 636 bp; *cob*, 811 bp), separate and combined, were performed with raxmlHPC-SSE3 (Stamatakis, 2014) using the GTR+I (*cob*), GTR+G (ITS, and combined data) models of substitution as determined by jModeltest v2.1.5 using AIC (Darriba *et al.,* 2012). Branch support was calculated by non-parametric bootstrapping using 500 pseudoreplicates.

References

Darriba D, Taboada GL, Doallo R, Posada D. 2012. jModelTest 2: more models, new heuristics and parallel computing. *Nature Methods* **9:** 772.

Katoh S. 2013. MAFFT multiple sequence alignment software version 7: improvements in performance and usability. *Molecular Biology and Evolution* **30:** 772–780.

Stamatakis A. 2014. RAxML version 8: a tool for phylogenetic analysis and post-analysis of large phylogenies. *Bioinformatics* **30:** 1313–1313.
